# Optimal Design of Miniaturized Reflecting Metasurfaces for Ultra**-**Wideband and Angularly Stable Polarization Conversion

**DOI:** 10.1038/s41598-018-25934-3

**Published:** 2018-05-16

**Authors:** Michele Borgese, Filippo Costa, Simone Genovesi, Agostino Monorchio, Giuliano Manara

**Affiliations:** Università di Pisa, Dipartimento di Ingegneria dell’Informazione, Pisa, Via Caruso 16, 56122 Italy

## Abstract

An ultra-wideband linear polarization converter based on a reflecting metasurface is presented. The polarizer is composed by a periodic arrangement of miniaturized metallic elements printed on a grounded dielectric substrate. In order to achieve broadband polarization converting properties, the metasurface is optimized by employing a genetic algorithm (GA) which imposes the minimization of the amplitude of the co-polar reflection coefficient over a wide frequency band. The enhanced angular stability of the polarization converter is due to the miniaturized unit cell which is obtained by imposing the maximum periodicity of the metasurface in the GA optimization process. The pixelated polarization converter obtained by the GA exhibits a relative bandwidth of 102% working from 8.12 GHz to 25.16 GHz. The analysis of the surface current distribution of the metasurface led to a methodology for refining the optimized GA solution based on the sequential removal of pixels of the unit cell on which surface currents are not excited. The relative bandwidth of the refined polarizer is extended up to 117.8% with a unit cell periodicity of 0.46 mm, corresponding to λ/20 at the maximum operating frequency. The performance of the proposed ultra-wideband polarization metasurface has been confirmed through full-wave simulations and measurements.

## Introduction

The manipulation of the electromagnetic wave polarization state is a desirable characteristic in several electromagnetic applications. Common applications are related to microwave communications and antennas^1^. They are also used to perform remote environmental monitoring^2^, or to realize microwave devices such as circulators and isolators^2^. Moreover, polarization converters are widely applied in optical instrumentation. Typically, they are realized with optical gratings and dichroic crystals^3^. A very effective way to manipulate several basic properties of the electromagnetic (EM) wave (amplitude, phase, polarization) is the use of metasurfaces. The latter are artificial surfaces designed to obtain particular electromagnetic or optical properties that cannot be found in nature. A metasurface is a single or multi-layer structure where periodic metallic elements with dimensions smaller than the operational wavelength are included. In this way, near the interface, anomalous reflections and refractions occur, which can be explained by the generalized Snell’s Law^4^. The scattering properties of the composite material can be manipulated through the choice of substrate material, the size and the geometry of the surface. Metasurfaces have been considered as an effective solution for the design of different devices and applications such as lens^5–8^, broadband low-profile antennas^9^, invisible cloak^10–14^, polarizers^15–17^, and super-resolution imaging^18^. In particular, metamaterials with negative permittivity and with negative refracting index have been employed for the design of novel superlenses^19,20^. Recently, polarization converters based on metasurfaces have been intensively studied because of their capability of manipulating the polarization of EM waves. Several examples of linear to linear^21,22^, linear to circular^23–25^ and multifunctional^26,27^ polarization converters have been proposed in the literature. In addition, different types of polarization converters based on anisotropic and chiral metasurface have been proposed^28–30^. However, these polarization converters suffer from a narrow bandwidth which could be a limitation in their practical application. Many studies have been devoted to techniques for the expansion of the bandwidth. A possible approach is to design reconfigurable metasurfaces which can be controlled with p-i-n^31^ or varactor^32^ diodes. An alternative approach is to stack periodic frequency selective surfaces in a multilayer configuration^33^, in which complementary circular symmetric split-ring resonators are employed in a multilayer structure. Another approach is to generate multiple plasmon resonances^34^ by electric and magnetic resonances. In this way, it is possible to achieve the bandwidth expansion of crosspolarization reflection on the metasurface^35^. Recently, metasurfaces based on galinstan^27^ and graphene^36^ have been proposed for the design of broadband polarization converters. In ref.^21^, a double V-shaped metasurface operating from 12.4 GHz to 27.96 GHz is printed on a FR4 substrate with a thickness of 1.6 mm. In this case, the expansion of the bandwidth is based on the generation of multiple EM resonances. This polarization converter can change a linearly polarized wave over a relative bandwidth of 77.1%. An ultra-wideband reflective linear cross-polarization converter based on anisotropic metasurface has been presented by Jia-Liang Wu *et al*.^37^. The unit cell is composed by a square-shaped resonator with diagonal and metallic ground sheet separated by dielectric substrate with a thickness of 3 mm. This polarization converter exhibits a relative bandwidth of 91.2% working from 7.57 GHz to 20.46 GHz.

The presence of the ground plane in reflective metasurfaces allows for the reflection of almost all electromagnetic energy impinging on the surface. For this reason, this kind of metasurface is employed for applications in which a high efficiency level is required. Polarization converters are employed in several applications in microwave regime. Specifically, in the field of microwave antennas, polarization converters are particularly desirable for the design of reflectarray antennas^38–40^ and leaky wave antennas^41^. In addition, this kind of devices are widely used for military applications in the microwave regime for the design of stealth surfaces. In these cases, wideband polarization converters (as the polarizer presented in the paper) are needed. In particular, the reduction of the Radar Cross Section (RCS) of targets relies on wideband reflecting polarization rotators^42–45^ thus exploiting the scattering diffusion phenomenon^44^. Polarization converting surfaces are also a fundamental building block in several microwave and optical devices^46–48^.

In this paper, an ultra-wideband polarization converter based on a single-layer reflective metasurface is presented. This device is able to perform a linear-to-linear polarization conversion for an orthogonal incidence of the EM wave. The aspects that most distinguishes this polarization converter from all others proposed in literature are its operative bandwidth and the compact size of the periodic elements. In fact, this polarization converter is able to work from 8.12 GHz to 25.16 GHz with a relative bandwidth of 102%. In addition, in order to further improve the performance of the polarization converter, the topology of the metasurface has been modified with a refinement procedure^49,50^. The relative bandwidth has been extended up to 117.8% with a unit cell periodicity of 0.46 mm, that is λ/20 at the maximum operating frequency. In addition, the miniaturized size of the unit cell allows to obtain robust polarization conversion performance with respect to the elevation angle of the impinging wave.

The paper is organized as follows. Section II shows the theoretical background of the scattering from a grounded metasurface and the working principle of the proposed polarization converter. In section III, the design and the refinement of the unit cell topology is addressed and simulated results are shown. Finally, in section IV the measures of the fabricated prototype are shown.

## Theoretical background

**Figure 1:**
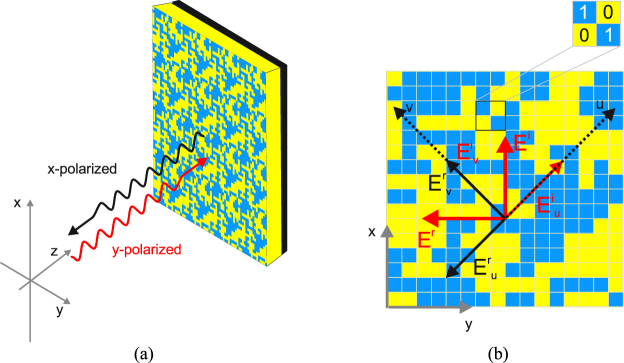
(**a**) Reflective linear polarization converter geometry. (**b**) Representation of the incident electric field decomposition in the *uv-*coordinate system.

The interaction mechanism of an EM wave impinging on an interface when the interface is formed by a periodic arrangement of metallic elements are regulated by generalized Snell’s law^4^. By properly designing the surface, it is possible to exploit the anomalous interaction of the EM wave with the interface in order to achieve a non-conventional behaviour of the reflecting surface. In particular, for a reflective linear polarization converter, the metasurface needs to reflect the two components of the impinging electric field with the same amplitude and with a phase difference equal to 180°. In Fig. 1a, the geometry of a reflective linear polarization converter is shown.

In this case, a *y*-polarized wave impinges orthogonally on the surface of the grounded periodic surface and the electric reflected field is directed along the *x*-axis. The working principle of the polarization converter can be explained by considering the additional coordinates system originated from the two perpendicular symmetric axes *u* and *v*, which are 45° rotated with respect to the *y*-axis shown in Fig. 1(b). It is therefore possible to decompose the *y*-polarized field into *u* and *v* components and to write the incident electric field as follows.

1 $${\overrightarrow{E}}^{inc}={E}_{0}\hat{x}={\overrightarrow{E}}^{inc}\cdot \hat{u}+{\overrightarrow{E}}^{inc}\cdot \hat{v}={E}_{0}\,\sin (\frac{\pi }{4})\hat{u}+{E}_{0}\,\cos (\frac{\pi }{4})\hat{v}$$

The reflected electric field can be written as a function of the reflection coefficients *R*_*u*_ and *R*_*v*_ in the *uv-*coordinates system:

2 $${\overrightarrow{E}}^{ref}={E}_{0}\hat{x}={\overrightarrow{E}}^{ref}\cdot \hat{u}+{\overrightarrow{E}}^{ref}\cdot \hat{v}={R}_{u}{\overrightarrow{E}}^{inc}\cdot \hat{u}+{R}_{v}{\overrightarrow{E}}^{inc}\cdot \hat{v}$$

Consequently, the reflected electric field is parallel to the *x-*axis only if the following conditions are met:

3 $${R}_{u}{\overrightarrow{E}}^{inc}\cdot \hat{u}=-\,{\overrightarrow{E}}^{inc}\cdot \hat{u}\Rightarrow {R}_{u}=-\,1$$

4 $${R}_{v}{\overrightarrow{E}}^{inc}\cdot \hat{v}=-\,{\overrightarrow{E}}^{inc}\cdot \hat{v}\Rightarrow {R}_{v}=1$$

On the basis of the conditions (3) and (4) it is possible to deduce the characteristics required to the metasurface. First of all, since *R*_*u*_ ≠ *R*_*v*_, the metasurface needs to be anisotropic. In addition, since the magnitude of *R*_*u*_ and *R*_*v*_ must be equal to 1.0, a dielectric substrate with a very low tangent loss is required.

The most challenging task of a wide-band polarization converter design is to find the proper combination of thickness, number of layers, substrate materials and unit cell topology of the metasurface matching the conditions (3) and (4) over a wide frequency range.

In order to provide a more comprehensive explanation of the working principle of a reflective metasurface, the effect of the ground plane will be analysed. Indeed, it is worth highlighting that the anisotropic behaviour of the reflecting surface does not depends only on the unit cell topology, but it is affected by the interaction between the periodic surface and the ground plane. In fact, treating the periodic arrangement of metallic elements as a partially reflecting surface, the working principle of a reflecting metasurface can be explained with the interference theory. In light of this, the backscatterd field can be interpreted as a sum of infinite contributions of electric field transmitted through the metasurface interface and reflected by the ground plane. In the following analysis, the co-polar reflection (transmission) coefficient for a *y*-polarized wave which travels from the medium 1 to the medium 2 is indicated as *R*_*yy12*_ (*T*_*yy12*_). Similarly, the cross-polar reflection (transmission) coefficient for a *y*-polarized wave which travels from the medium 1 to the medium 2 is *R*_*yy12*_ (*T*_*yy12*_). In this analysis, the free space is referred as medium 1 and the dielectric substrate is referred as medium 2. The propagation constant inside the dielectric substrate with electric permittivity ε is $\beta =\sqrt{\varepsilon }{k}_{0}d$ (*k*_0_ = free space propagation constant and *d* is the substrate thickness). When the EM wave impinges on the interface, it is partially reflected and partially transmitted. The transmitted wave travels in the dielectric substrate and is reflected by the ground plane, which is characterized by a reflection coefficient equal to −1. When the two waves impinge on the interface, they have experienced a phase rotation equal to ${e}^{j2\beta }$. At the air/metasurface interface, the *y*-polarized impinging field can be immediately reflected in cross-polarization by the metasurface but some of the signal penetrates the first layer and start bouncing between the metasurfaces and the ground plane. The part of the signal which penetrates the first interface can have two polarization states (*x* and *y* polarization). This signal can emerge in cross-polarization if suitable combinations of reflections verifies. For instance, the signal can penetrate the first layer remaining y-polarized, but it can be converted as it impinges the metasurface from the bottom. Equivalently, the transmitted signal can penetrate the first interface being converted in x-polarization and emerge after one bounce in the ground plane. It can be demonstrated that an infinite set of these contributions exist and can sum up coherently or not. Each of these terms can be written in the form of geometric series. Beside the rigorous analytical analysis, it is worth underlining that the total reflection of the composite surface can be written as a summation of terms of different orders. The number of terms needed to correctly estimate the backscattered field depends on the reflectivity of the surface. In the case of metasurface reported in Fig. 1, the cross-polar scattered field has been estimated considering all terms from zero-th order (*R*_*yy12*_) up to fourth order $({\tilde{R}}_{yy}^{4})$. In this way, the co-polar and cross-polar component of the scattered field can be expressed as follows:

5 $${\tilde{R}}_{yy}\approx {R}_{yy12}+{\tilde{R}}_{yy}^{1}+{\tilde{R}}_{yy}^{2}+{\tilde{R}}_{yy}^{3}+{\tilde{R}}_{yy}^{4}$$

6 $${\tilde{R}}_{yx}\approx {R}_{yx12}+{\tilde{R}}_{yx}^{1}+{\tilde{R}}_{yx}^{2}+{\tilde{R}}_{yx}^{3}+{\tilde{R}}_{yx}^{4}$$

**Figure 2:**
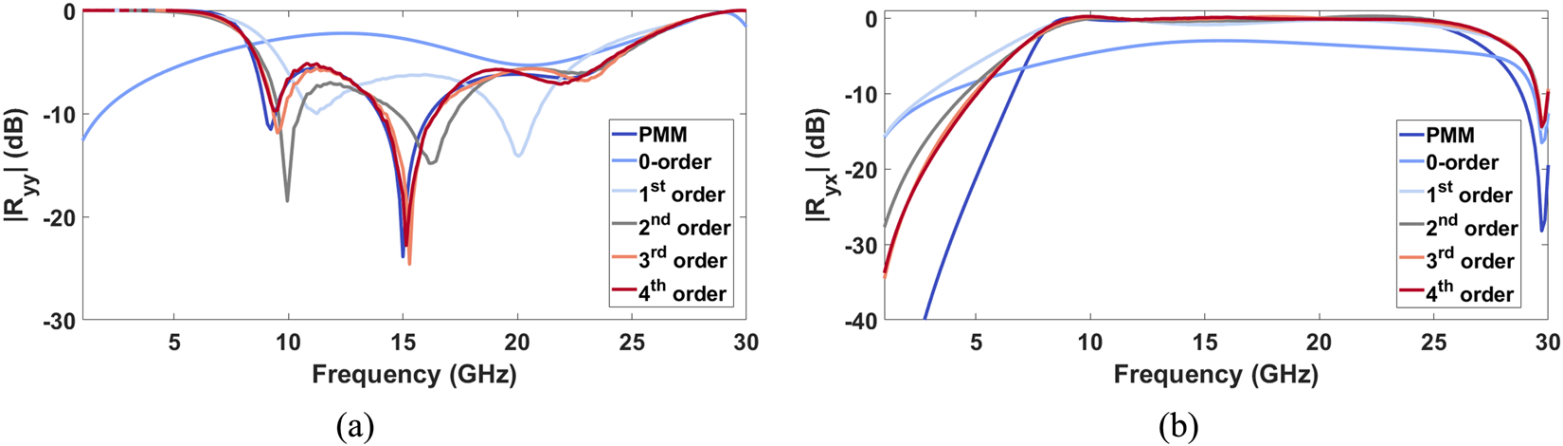
(**a**) *|R*_*yy*_*|* and (**b**) *|R*_*yx*_*|* as a function of the frequency for the optimized unit cell calculated by the PMM and with the interference theory approach extended up to the fourth order.

The co-polar and cross-polar reflection coefficient of the optimized unit cell calculated by using an in-house developed Periodic Method of Moments code (PMM)^51^ and with the interference theory approach have been respectively reported in Fig. 2(a,b).

The *R*_*yy21*_ and *R*_*yx21*_, as a function of the frequency for the optimized unit cell calculated with the PMM and with the interference theory approach extended up to the fourth order, are shown in Fig. 2(a,b), respectively.

From both figures, it is evident that increasing the order of the interference theory analysis, the agreement with the PMM calculated curve increases. In particular, considering only the zero-th order term (metasurface without ground plane), the deep nulls of the co-polar reflection are not present. This analysis highlights as the desired operation of the polarization converter is obtained when the interaction between the metasurface and ground plane is controlled.

## Design process

The aim of this work is to design a linear polarization converter based on a reflective metasurface. As shown in the previous section, the design of the device is based on the choice of the metasurface parameters, which are able to satisfy the conditions (3) and (4) over a wide frequency band. A possibility to design these kind of devices is to use canonical shapes. However, in the latter case is difficult to enlarge the bandwidth above 90% and preserve at the same time a compact unit cell size. Indeed, if the unit cell is not sufficiently compact, grating lobes can emerge at the highest operation frequency of the wideband polarization converter. For this reason, a numerical code based on a genetic algorithm (GA) has been employed for the design of the metasurface^52–54^.

The basic periodic cell is discretised in a 16 × 16 binary pixel matrix in which 1 or 0 represents whether a pixel is metallized or not. The GA selects the topology of the pixel matrix on the basis of the objective function defined as consists in minimizing the reflection coefficient of the fundamental Floquet harmonic over a number of frequency points within the desired frequency band. The goodness of the solution is evaluated by using the following fitness function calculated on *n*_*f*_ = 21 frequency points:

7 $$Fit=\sum _{n=1}^{{n}_{f}}{|{R}_{xx}^{i}|}^{2}+{|{R}_{yy}^{i}|}^{2}$$

In order to ensure a fast convergence of the algorithm, the initial population is obtained from the best chromosomes of several trial populations. The following generations are created by selecting only new chromosomes which present fitness values lower than the older chromosomes. At least two metallized contiguous pixels are needed in order to assign a roof-top basis function. In this work, a specific formulation of the GA based on the weighted roulette wheel selection scheme is applied to the design of the metasurface in combination with the PMM code. At the end of the optimization process, the unit cell topology is cleaned from isolated pixels on which is not possible to define a basis function. Afterwards, a refining procedure is run in order to further optimize the polarization reflecting surface and checking the usefulness of the unit cell pixels. The GA algorithm is run about 15 time and each GA run required roughly 4 hours. The GA is stopped after 200 generations or if the fitness function is stable for more than 50 generations. The estimated total computational time is of the order of two or three days (it mainly depends on the number of times that the GA is run). Being a stochastic process, it is not guaranteed that the global best solution is found with a precise criterion but, for the best of our experience, running the GA algorithm more than 15 times does not provide a better solution than the one selected with the first 15 runs. On the other hand, the refinement process based on the sequential removal of the pixels of the unit cell required few minutes.

The main advantage of the Periodic Method of Moments (PMM) with respect to other fullwave methods, relies in the computational time required to the simulation of a Frequency Selective Surface. In particular, the PMM is able to perform a full-wave simulation of a very complex structure (e.g. multilayer stackup composed of multiple lossy dielectric and FSS layers) over of hundreds of frequency points in few minutes.

**Figure 3:**
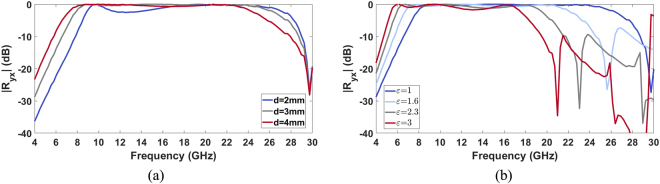
|Ry_x_| as a function of the frequency for an air substrate of thickness *d* = 2 mm, 3 mm, 4 mm (**a**); |*R*_*yx*_| as a function of the frequency for a loss less substrate with a thickness of 3 mm for ε = 1, 1.6, 2.3, 3 (**b**).

As demonstrated in the previous section, in order to convert the polarization of the impinging electric field, a low loss dielectric substrate is needed. For this reason, in the optimization process of the unit cell, an air spacer has been employed as substrate of the metasurface. In order to obtain a polarization converter with stable performance with respect to the elevation angle, a unit cell of miniaturized dimensions is required. For this purpose, in the GA optimization process, the maximum periodicity of the metasurface is imposed. In Fig. 1(b), the unit cell topology obtained at the end of the optimization process is shown. The *x, y*-periodicity (*T*) chosen by the GA is equal to 4.6 mm and the thickness *d* is equal to 3 *mm*. Once the unit cell geometry has been selected, a parametric analysis has been performed in order to optimize the thickness of the air layer. In Fig. 3a, the cross-polar reflection coefficient (*R*_*xy21*_) as a function of the frequency is shown for the values of the substrate thickness *d* equal to 2 mm, 3 mm and 4 mm. As it is evident from Fig. 3(a), with the optimal value *d* = 3 mm, the reflection coefficient *R*_*xy21*_ is higher than −1dB over the frequency band ranging from 8.12 GHz to 25.16 GHz. The operative bandwidth is 17.04 GHz, which corresponds to a relative bandwidth equal to 102%. With a thinner substrate, a 700 MHz bandwidth reduction occurs at low frequency. Moreover, the curve drops below −1dB around 12.5 GHz. Conversely, by increasing the substrate thickness, a reduction of the bandwidth at high frequency is present. In particular, when *d* = 4 mm, the highest working frequency is reduced to 23 GHz. These considerations justify the choice *d* = 3 mm. With a substrate thickness equal to 3 mm, the performance of the metasurface has been analysed for different permittivity values of the dielectric substrate. In Fig. 3(a), the reflection coefficient *R*_*xy21*_ as a function of the frequency is shown for the values ε of the losses less substrate equal to 1, 1.6, 2.3 and 3. As shown in the figure, increasing the dielectric permittivity, the highest working frequency of the polarization converter decreases.

**Figure 4:**
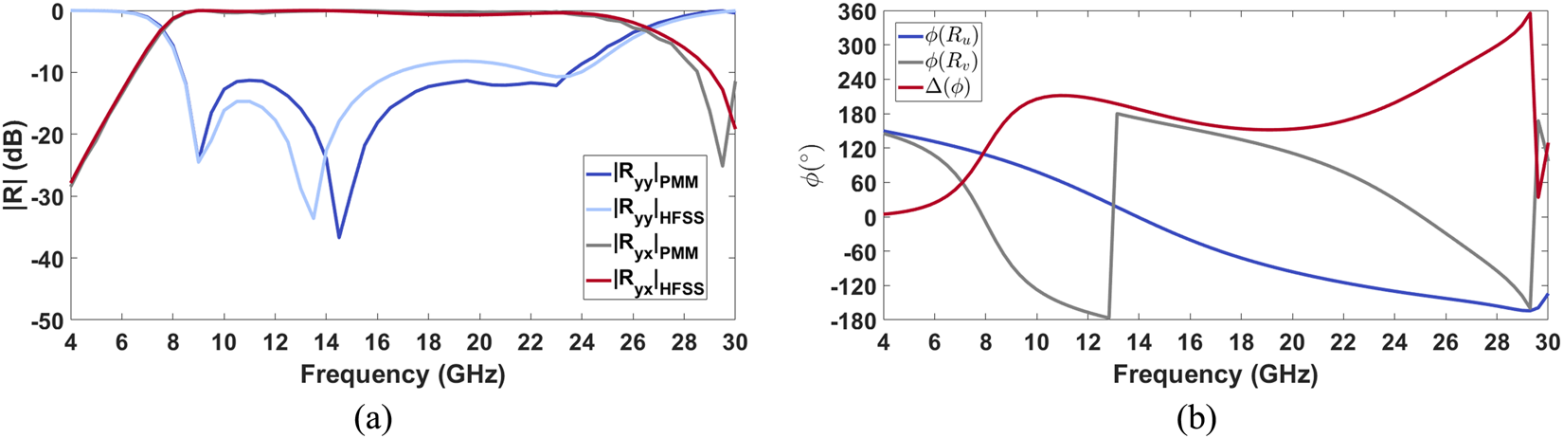
|Ry_*y*_| and |*R*_*yx*_| as a function of the frequency calculated by the PMM and by HFSS (**a**); (**b**) Phase of the reflection coefficient *ϕ*(*R*_*u*_) and *ϕ*(*R*_*v*_) and phase difference Δ(*ϕ*) = *ϕ*(*R*_*u*_) − *ϕ*(*R*_*v*_) as a function of the frequency calculated by the PMM.

In the light of the presented parametric analysis, an air dielectric substrate with a thickness of 3 mm has been adopted in the final design of the metasurface. In order to evaluate the performance of the proposed design, full-wave simulations have been performed by the PMM and by Ansys Electronic v. 16 (HFSS). In Fig. 4(a), the reflection coefficients *R*_*yy21*_ and *R*_*yx21*_ calculated by the PMM and by HFSS are shown. It is apparent that there is a good agreement between the two numerical methods in the evaluation of the copular reflection coefficient. The simulations show a relative operative bandwidth of the polarization converter equal to 102%. A shift between the co-polar reflection coefficients provided with the two numerical methods is revealed between 8 GHz and 23 GHz. On the contrary, there is good agreement on the bands 4 GHz-8 GHz and 23 GHz-30 GHz. Moreover, the phase of the reflection coefficient along the *u* and *v* directions (*ϕ*(*R*_*u*_), *ϕ*(*R*_*v*_)) and phase difference Δ(*f*) = *ϕ*(*R*_*u*_) − *ϕ*(*R*_*v*_) as a function of the frequency calculated by the PMM are reported in Fig. 4(b).

**Figure 5:**
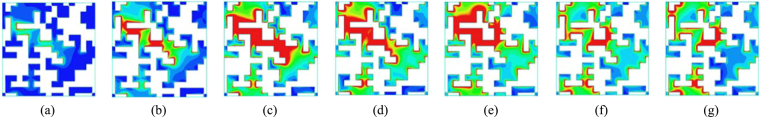
Surface current distribution of the unit cell obtained at the end of the optimization process calculated at 4 GHz (**a**), 6 GHz (**b**), 8 GHz (**c**), 16 GHz (**d**), 26 GHz (**e**), 34 GHz (**f**) and 36 GHz (**g**).

In order to better understand the physical mechanism of the metasurface obtained at the end of the optimization process, the surface currents distributions on the unit cell are presented. In Fig. 5, the current distributions calculated by HFSS at 4, 6, 8, 16, 26, 34 and 36 GHz are shown.

### Refining procedure

**Figure 6:**
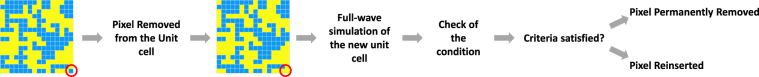
Refinement process of the unit cell topology.

As mentioned in the previous section, the proposed polarization converter operates from 8.12 to 25.16 GHz. As it is evident from Fig. 5(a,b), beyond the operative band, surface currents are not excited on the unit cell. Conversely, within that working band, intense currents flow on the metasurface (Fig. 5(c–e)). It is interesting to analyse the behaviour of the unit cell at frequencies higher than the working bandwidth, for example at 34 GHz and 36 GHz. It is evident from Fig. 5(f,g) that some parts of the unit cells support strong surface currents but most of the pixels are not excited by the currents. In addition, it is important to observe that there are metalized pixels of the unit cell on which the surface currents are not excited for any frequency. This suggests that not all the pixels of the unit cell are needed to convert the polarization of the impinging EM wave. Consequently, some pixels can be removed without compromising the performance of the metasurface. Therefore, the unit cell provided by the GA can be further refined by removing the unnecessary pixels. The refinement process of the metasurface’s topology is described in Fig. 6.

Firstly, one pixel is removed from the unit cell provided by the GA and a full-wave simulation of the new unit cell is performed by the PMM code. In order to understand whether the pixel removed is necessary or not to the metasurface, the reflection coefficient ${\tilde{R}}_{yx}$ calculated by the full wave is compared against the cross-polar reflection coefficient of the original unit cell *R*_*yx*_. In particular, by indicating with Δ the value of a fixed threshold, the single pixel is removed on the basis of the following condition:

8 $$\sum _{i=1}^{{n}_{f}}|{\tilde{R}}_{yx}-{R}_{yx}|\le {\rm{\Delta }}$$

**Figure 7:**
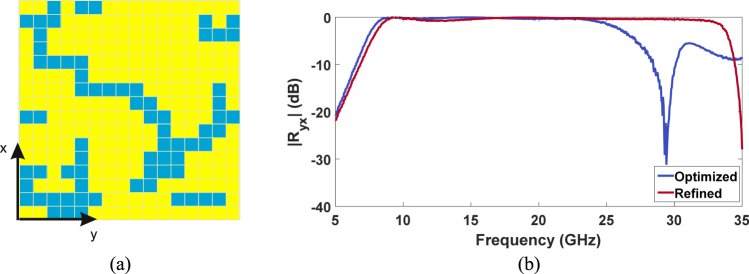
(**a**) Refined unit cell; (**b**) |*R*_*yx*_| as a function of the frequency for the original and refined unit cell.

For each one of the *n*_*f*_ frequency points, the magnitude of the difference between the two reflection coefficients is calculated. If the sum of the difference is above Δ, it means that the polarization conversion capabilities of the metasurface are compromised by the removal of the pixel. Consequently, the pixel is re-inserted in the unit cell. Conversely, if the difference is above the threshold, the pixel is permanently removed. This procedure is applied to each pixel of the original unit cell. In the case of the proposed polarization converter, the threshold was set to 0.1 dB. The unit cell topology obtained at the end of the refinement process is reported in Fig. 7(a).

In Fig. 7(b) the cross-polar reflection coefficient |*R*_*yx*_| as a function of the frequency for the optimized and refined unit cell is shown. The highest working frequency of the refined polarization converter is extended up to 32.84 GHz. On the other hand, a 370 MHz increase of the lowest working frequency has found thus shifting the starting working frequency to 8.49 GHz. The final effect of the refinement process is a 15.8% increment of the relative bandwidth thus resulting in 117.8% overall bandwidth. It is worth underlining that the topology of the refined unit cell is highly dependent on the value of Δ. In particular, the stricter the condition on the tolerable error the lower is the number of removed pixels.

**Figure 8:**
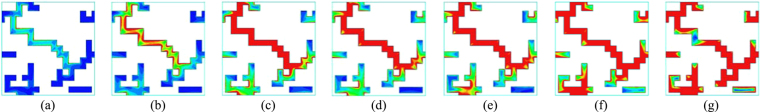
Surface current distribution of the refined unit cell at 4 GHz (**a**), 6 GHz (**b**), 8 GHz (**c**), 16 GHz (**d**), 26 GHz (**e**), 34 GHz (**f**) and 36 GHz (**g**).

The surface currents have been calculated for the refined unit cell at 4, 6, 8, 16, 26, 34 and 36 GHz and are reported in Fig. 8.

As in the case of the original unit cell topology, the surface currents are not excited outside of the working band of the polarization converter (Fig. 8(a,b)). Differently from the case reported in Fig. 5, there are no pixels that are not excited by the surface current in the working band. Moreover, it should be noted that an intense surface currents distribution is supported by the refined unit cell also at 34 GHz and 36 GHz. Unlike the case reported in Fig. 5(f,g), all the pixels of the unit cell are supporting the surface currents with a consequent expansion of the operative bandwidth of the metasurface. The pixels removed from the original unit cell deteriorated the polarization conversion capability of the metasurfaces at high frequency.

In order to have an easier and more complete overview of the state of the art, the performance and the geometry in terms of the most remarkable examples of reflective linear polarization converter presented in literature have been reported in Table 1.

**Table 1:** Comparison of the performance of ultra-wideband liner polarization converters.

Polarization Converter	ε_r_	Thickness d (mm)	d/λ_in_	Periodicity (T)	T/λ_in_	f_in_ (GHz)	f_end_ (GHz)	Bandwidth_−1dB_ (GHz)	Bandwidth_−1dB_ (%)
Our solution - GA	1.05	3	0.08	4.6	0.13	8.12	25.16	17.04	102
Our solution - refined	1.05	3	0.09	4.6	0.13	8.49	32.84	24.35	117.8
ref.^42^	4.3	4	0.15	10	0.39	5.71	15.02	9.31	89.82
ref.^21^	4.4	1.6	0.14	6.4	0.55	12.4	27.96	15.56	77.1
ref.^37^	2.2	3	0.11	11.5	0.43	7.57	20.46	12.89	91.2
ref.^56^	2.65	3	0.16	10	0.54	10	18.4	8.4	59.2

**Figure 9:**
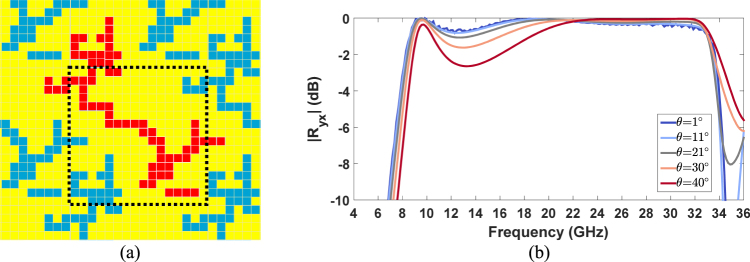
Metasurface’s topology: the resonant element (highlighted in red) extends beyond the minimum period of the metasurface (black dotted line) (**a**); Simulated |*R*_*yx*_| of the refined metasurface as a function of the frequency for different elevation angles (*θ* = 1°, 11°, 21°, 30°, 40°) of the incident wave (**b**).

The proposed polarization converter is highly miniaturized. Its periodicity *T* is at least 50% smaller than the average periodicity of the other analysed solutions. The miniaturization of the proposed metasurface is evident in Fig. 9(a) where the topology of the metasurface is shown. In this figure it is noticeable that the resonant element (highlighted in red) extends beyond the minimum period of the metasurface (black dotted line) thus leading to a miniaturized metasurface metasurface^55^.

The direct consequence of the miniaturized topology is the polarization conversion stability of the metasurface with respect to the elevation angle of the incoming wave. This is evident in Fig. 9(a) where the simulated |*R*_*yx*_| of the refined metasurface as a function of the frequency for different elevation angles (*θ* = 1°, 11°, 21°, 30°, 40°) of the incident wave is shown.

The numerical results demonstrate that the polarization converter exhibits robust performance up to *θ* = 40° and there is no grating lobe onset in the operating frequency range even at oblique incidence because of the unit cell miniaturization. In addition, the proposed solution exhibits smallest thickness (*d/*λ_*in*_) with respect to the wavelength at the lower working frequency (λ_*in*_). Moreover, it is remarkable to say that the relative bandwidth of 117.8% of the best solution is higher than any other similar device presented in the literature so far.

### Fabricated Prototype and Measured results

In order to validate the numerical results presented in the previous section, some prototypes have been fabricated and measured. As shown in Table 1, the GA-optimized polarization converter works up to 25.16 GHz whilst the refined polarization converter works up to 32.84 GHz. The highest working frequency of the available vector network analyzer (Keysight E5071C) is 20 GHz. For this reason, in order to validate the proposed designs, a scaled version of the GA-optimized and refined polarization converters have been designed thus obtaining a downshift of the working bandwidth. In particular, the polarization converts have been scaled by 50% resulting in a substrate thickness equal to 6 mm and a periodicity *T* of 9.2 mm.

**Figure 10:**
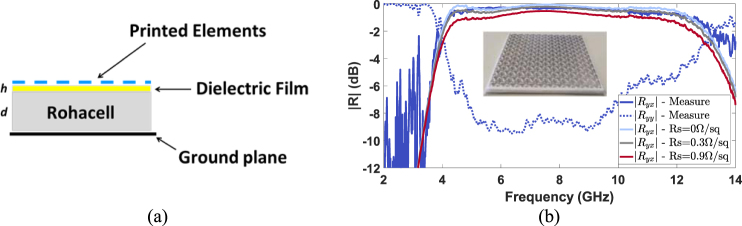
(**a**) Stack-up of the polarization converter. (**b**) Preliminary prototype: comparison between the measured |*R*_*yy*_| and |*R*_*yx*_| and the simulated |*R*_*yx*_| as a function of the frequency for the for different values of the surface resistance R_s_ = 0, 0.3, 0.9 Ω/sq.

With the purpose of validating the working principle of the proposed polarization converter, a preliminary prototype has been fabricated. The resonant elements are is inkjet-printed on a thin sheet of NB-TP-3GU100 Mitsubishi paper. The silver nanoparticle ink is deposited with a conventional piezoelectric inkjet printer (Brother DCP-J152W). The thin dielectric film with thickness *h* = 150 μm is accommodated on a layer of Rohacell HF31 (ε_r_ = 1.05 − j0.0017) with thickness *d* = 6 mm. As shown in Fig. 10(a), the Rohacell HF31 layer is backed by a metallic ground plane.

**Figure 11:**
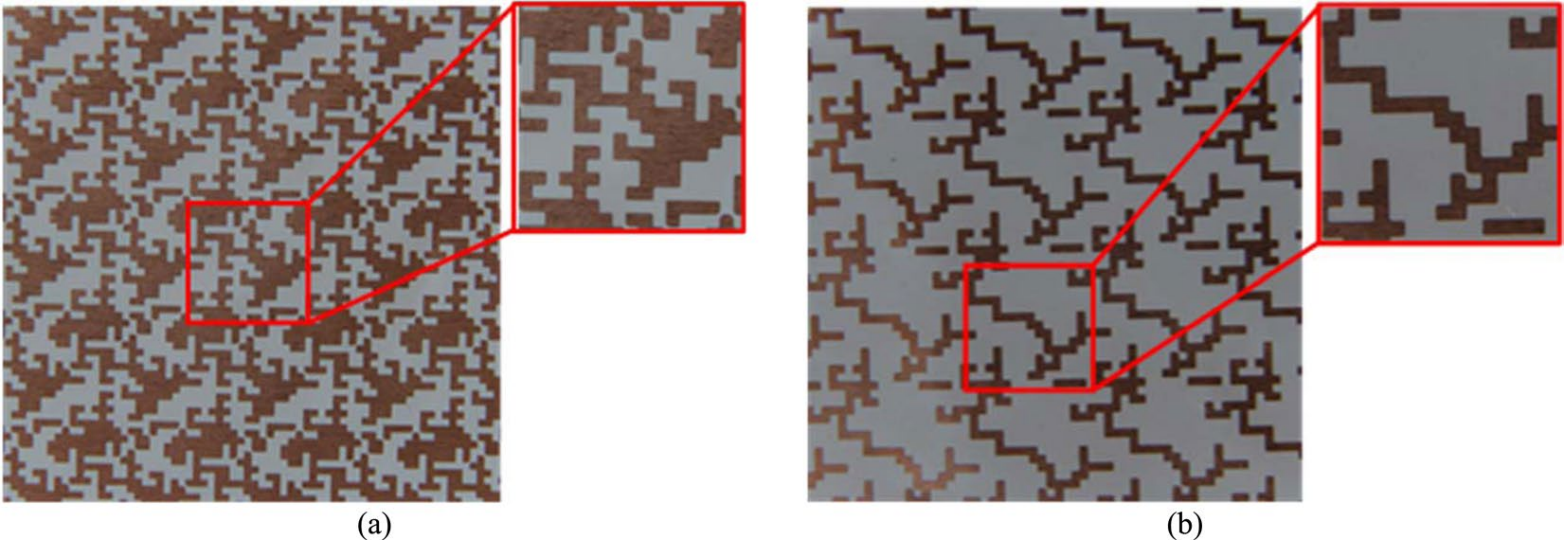
GA-optimized (**a**) and refined (**b**) prototype fabricated with the photolithographic process. The zoom view of the unit cell is shown in the inset.

As shown in Fig. 10(b) the preliminary prototype exhibits a relative bandwidth of 101.3% working from 4.16 GHz to 12.7 GHz. This value relative bandwidth is in agreement with the expected performance of the GA-optimized polarizer reported in Table 1. Moreover, the measured performance of the preliminary prototype has been compared to numerical simulation for different values of the surface resistance of the printed elements. Figure 10(b) shows a good agreement between measurements and simulated |*R*_*yx*_| when R_s_ = 0.3 Ω/sq. It has to be underlined that there is no dramatic degradation of the performance even if non-perfect metallic pattern is employed in the design of the polarization converter. The GA-optimized and refined polarization converter has been also fabricated with the photolithographic process. The adopted stack-up is the same of the one shown in Fig. 10(a) but the dielectric film is a layer of Kapton (ε_r_ = 3.5-j0.007) with thickness *h* = 75 μm. The GA-optimized and refined prototype fabricated with the photolithographic process are shown in Fig. 11(a,b), respectively. The zoom view of the unit cells for the GA-optimized and refined prototype is shown in the insets of Fig. 11.

**Figure 12:**
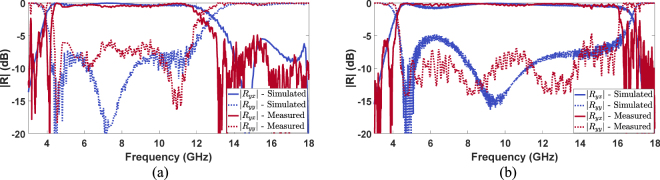
Comparison between the simulated and measured |*R*_*yx*_| and |*R*_*yy*_| as a function of the frequency for the GA-optimized (**a**) and refined (**b**) polarization converter.

The simulated and measured |*R*_*yx*_| and |*R*_*yy*_| as a function of the frequency for the GA-optimized and refined polarization converter are reported in Fig. 12(a,b). The measured results of the GA-optimized polarization converter show a relative bandwidth of 93.37% working from 4.3 GHz to 11.83 GHz (Fig. 12(a)) while the measured results of the optimized polarization converter show a relative bandwidth of 115.6% working from 4.3 GHz to 16.08 GHz (Fig. 12(b)). The simulated and measured performance of the e scaled prototypes fabricated with the photolithographic process are summarized in Table 2.

**Table 2:** Summary of the simulated and measured performance of the scaled prototypes fabricated with the photolithographic process.

Polarization Converter	f_in_ (GHz)	f_end_ (GHz)	Bandwidth_−1dB_ (GHz)	Bandwidth_−1dB_ (%)
GA-optimized Simulation	4.1	12.29	8.19	99.94
GA-optimized Measure	4.3	11.83	7.53	93.37
Refined Simulation	4.3	16.34	12.04	116.67
Refined Measure	4.3	16.08	11.78	115.6

## Conclusion

An optimization procedure for the design of ultra-wide band reflecting metasurfaces for polarization conversion has been presented. The pixelated unit cell topology has been optimized with a GA in conjunction with a full-wave code based on a Periodic Method of Moments. With the aim of obtaining a polarization converter with robust performance with respect to the elevation angle of the impinging wave, a miniaturized metasurface is designed. The miniaturized unit cell is obtained limiting the maximum periodicity of the metasurface in the optimization process. The polarization converter obtained at the end of the optimization process exhibits a relative bandwidth of 102%. From the analysis of the surface current distribution it was observed that some pixels of the unit cell were unnecessary. Consequently, an innovative approach aimed at removing the useless pixels have been proposed. The polarization converter obtained at the end of the refinement process exhibits a relative bandwidth of 117.8%. The numerical results are confirmed by measurement performed on scaled prototypes of both optimized and refined polarization converter.

## Methods

The measurements of the cross-polar reflection coefficient of the metasurfaces have been performed with one dual-polarized horn antenna (Flann DP240) operating between 2 GHz and 18 GHz. The adopted VNA is the Keysight ENA E5071C operating up to 20 GHz. The VNA is connected to the horn antenna with a Huber-Suhner cable (SUCOFLEX_102) with length equal to 2 m. The cable introduces acceptable losses up to 18 GHz. The measurements have been performed at normal incidence by using a single horn antenna.
